# Evaluation of concomitant peripheral arthritis in patients with recent onset axial spondyloarthritis: 5-year results from the DESIR cohort

**DOI:** 10.1186/s13075-019-1927-6

**Published:** 2019-06-06

**Authors:** Clementina López-Medina, Maxime Dougados, Adeline Ruyssen-Witrand, Anna Moltó

**Affiliations:** 1 Rheumatology Department, Cochin Hospital, 27 rue du Faubourg Saint Jacques, 75014 Paris, France; 2 Inserm (U1153), Clinical Epidemiology and Biostatistics, PRES Sorbonne Paris-Cité, Paris, France; 3 University of Córdoba, Córdoba, Spain; 4 Rhumatologie et Immunologie Clinique, CHU Purpan, Toulouse, France

**Keywords:** Axial spondyloarthritis, Peripheral arthritis, Clinical presentation

## Abstract

**Background:**

(a) To describe the prevalence and incidence of peripheral arthritis during 5 years of follow-up in recent axial spondyloarthritis (axSpA), (b) to evaluate factors associated with their appearance and (c) to assess their impact on treatment, patient-reported outcomes and sick leave after follow-up.

**Methods:**

Data from the early axSpA patients from the DESIR cohort (first 5 years of follow-up) were analysed. Prevalence and incidence of peripheral arthritis at each study visit were calculated. A multivariate analysis was performed to evaluate baseline factors associated with the development of the arthritis. The use of drugs, the impact on patient-reported outcomes and days of sick leave were compared in both groups over time.

**Results:**

Out of the 708 patients included in DESIR, 255 (36.0%) showed at least one episode of arthritis (151 before the inclusion visit and 104 during the follow-up), with an incidence of 3.7 cases per 100 person-years. Patients with peripheral arthritis were more likely (OR, 95%CI) to be aged ≥ 33 years (1.60, 1.12–2.27), non-smokers (1.58, 1.10–2.27) and HLAB27 negative (1.47, 1.04–2.08) and have presented with at least one episode of dactylitis (8.50, 4.96–14.60) and enthesitis (2.00, 1.41–2.84). Patients with peripheral arthritis showed a significant greater use of TNFb, csDMARDs and corticosteroids over follow-up; higher levels on BASDAI (40.46 vs. 34.28) and BASFI (27.89 vs. 22.52); poorer quality of life; and higher number of days of sick leave (17.97 vs. 12.78) over time.

**Conclusion:**

In recent axSpA, 36% of patients reported peripheral arthritis at any time of the disease, being associated with negative HLAB27, non-smokers and with other peripheral manifestations. Patients with arthritis showed a higher burden of disease.

## Background

Spondyloarthritis (SpA) is a chronic inflammatory disease that affects the axial skeleton, peripheral joints and enthesis [1]. SpA has been classically divided into subtypes, such as ankylosing spondylitis (AS), psoriatic arthritis (PsA), reactive arthritis, SpA associated with inflammatory bowel disease (IBD) and undifferentiated SpA. However, in 2009, the publication of the ASAS classification criteria allowed us to classify SpA patients in phenotypes, differentiating axial—axSpA—and peripheral SpA—pSpA [2]. Although spinal inflammation and sacroiliac inflammation are the hallmarks of axSpA, many of these patients present peripheral manifestations such as arthritis, enthesitis and dactylitis [1].

Arthritis and enthesitis are the most common peripheral features in axSpA and can be found predominantly in the lower limbs [1]. The prevalence of peripheral arthritis has been well described in AS patients, with percentages ranging between 22 and 30% [3]. However, the prevalence of this manifestation in the whole group of axSpA varies between the different cohorts. In recent-onset axSpA cohorts, such as DEvenir des Spondylarthropathies Indifferenciées Récentes (DESIR) and the SPondyloaArthritis Cught Early (SPACE), the prevalence of this symptom at baseline is 21.3% and 15%, respectively [4, 5]. However, non-recent axSpA cohorts, such as the GErman Spondyloarthritis Inception Cohort (GESPIC) and the South Swedish Arthritis Treatment Group (SSATG) register, reported higher percentages of peripheral arthritis at baseline in both radiographic and non-radiographic axSpA (37.4% and 40.9% in the GESPIC cohort; 50.0% and 45.0% in the SSATG register, respectively) [6, 7], demonstrating an increase in the prevalence of arthritis over time. However, the majority of these data refer only to baseline visits, and very few studies evaluate the time of onset of this clinical feature during follow-up. Some randomized controlled trials have assessed changes in peripheral joint count over time [8–11]; however, these studies did not evaluate the natural history of this clinical feature in daily care. For this reason, the DESIR cohort provided us the opportunity to study peripheral arthritis in recent axSpA patients within the framework of usual clinical practice.

The aims of the present study were (a) to describe the prevalence and incidence of peripheral arthritis in recent axSpA patients during 5 years of follow-up, (b) to describe the time of onset of peripheral arthritis with regard to the onset of axial symptoms, (c) to evaluate factors associated with their appearance and (d) to assess the impact of this clinical feature on treatment, patient-reported outcomes (PROs) and days of sick leave after 5 years of follow-up.

## Methods

### Patients

For this study, we used the data collected during the first 5 years of follow-up in the DESIR cohort (NCT01648907). The DESIR cohort has been previously extensively described [3]. Briefly, a total of 708 patients aged > 18 and < 50 years old from 25 centres in France with early inflammatory back pain (IBP) (> 3 months but < 3 years) based on either the Calin [12] or Berlin [13] criteria, suggestive of axSpA, were included. Previous biologic treatment was an exclusion criterion of the cohort; therefore, none of these patients were under biologic agent therapy at baseline. Visits were scheduled every 6 months during the first 2 years and yearly thereafter. The study fulfilled good clinical practice guidelines and was approved by the “Comité pour la Protection des Personnes Physiques (CPP) Île de France-III” ethical committee. All patients provided their written informed consent.

### Data collection

A case report form (CRF) was used to collect the following data during a patient interview at each centre.

- *Sociodemographic*: data regarding age, gender, ethnicity (Caucasian or non-Caucasian), profession (white or blue collar), level of education (university or non-university studies), days of sick leave and smoking and alcohol status were collected.
- Disease characteristics: data regarding HLA-B27 status, date of onset of IBP, family history of SpA, radiographic sacroiliac joint structure damage according to the modified New York criteria [14], sacroiliac inflammation on MRI according to the ASAS definition [15], abnormal C-reactive protein (CRP), enthesitis, dactylitis, uveitis, psoriasis and IBD were collected.
- PROs: the scores from the Bath Ankylosing Spondylitis Disease Activity Index (BASDAI) [16], the Bath Ankylosing Spondylitis Functional Index (BASFI) [17] and the SF-36 questionnaire [18] were evaluated and considered as continuous variable.
- Treatment: for data regarding treatment intake during the follow-up period, we collected the use of NSAIDs, TNF blockers (TNFb), csDMARDs, corticosteroids (oral, intramuscular and intravenous) and the number of corticosteroid intraarticular injections.
- Peripheral arthritis: The presence of peripheral arthritis was identified by the rheumatologist in two ways: (a) at the time of inclusion in the DESIR cohort, based on the history of peripheral arthritis prior to inclusion, and (b) at 0, 12, 18, 24, 36, 48 and 60 months via clinical exam, by the identification of synovitis. We also considered patients to have arthritis if they received intraarticular injections of corticosteroids at any time during follow-up. Neither arthralgia without synovitis nor periarticular injection of corticosteroids were considered as arthritis. The date of the first episode of arthritis (before inclusion or during follow-up) was collected for each patient.

### Statistical analysis

The prevalence (number of patients with the first episode of arthritis before inclusion) and incidence (number of patients with the first episode of arthritis during follow-up) were estimated, as well as the person-time rate during the 5 years of follow-up.

The time of onset of peripheral arthritis regarding axial disease (i.e. IBP) and regarding date of axSpA diagnosis were evaluated. In this way, patients were divided into three groups: peripheral arthritis before, concomitantly, or after axial symptom onset, as well as before, concomitantly, or after axSpA diagnosis.

We explored baseline demographic and clinical factors associated with peripheral arthritis at any time by univariate analysis and thereafter by multivariate logistic regression, including variables selected by the univariate analysis in the model (when *p* ≤ 0.20). Interactions and confounding factors were tested, and all comparisons were bilateral considering *p* ≤ 0.05 as a significant result.

Finally, we explored the impact of peripheral arthritis on treatment modalities, disease severity (BASDAI and BASFI), quality of life (SF-36 questionnaire) and days of sick leave over 5 years by mixed models with random effects (subject), including “arthritis” as the fixed independent variable. Mixed models adjusted for TNFb and csDMARDs use were also explored.

The data were analysed using R 3.2.3 version.

### Handling of missing data

There was no missing information at baseline. Longitudinal missing outcomes were handled by mixed-model estimation.

## Results

### Prevalence and incidence of peripheral arthritis

**Fig. 1:**
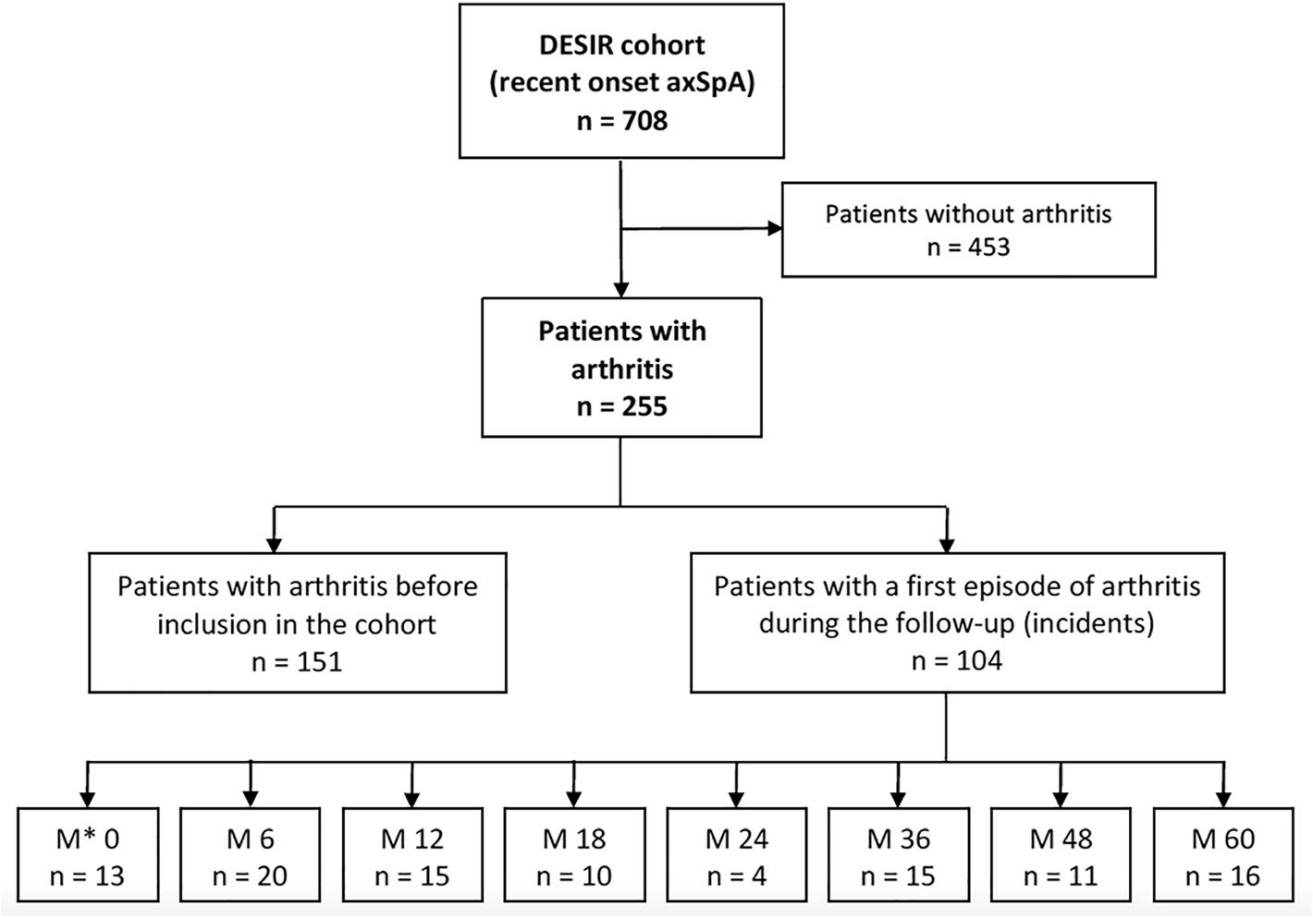
Flow chart with regard to the presence of concomitant peripheral arthritis in patients with recent onset axial spondyloarthritis. M, month of follow-up after the DESIR cohort baseline visit; axSpA, axial spondyloarthritis

Out of the 708 patients included in the analysis, a total of 255 [36.0% (95%CI 32.6–39.6)] patients suffered from peripheral arthritis at any time during the disease course (Fig. 1). Among these, 21.3% (151/708) had a prevalent arthritis (before the inclusion visit), and 18.7% (104/557) had an incident first episode of arthritis during the follow-up. The person-time rate was 3.7 new cases per 100 person-years, being the knee the most commonly joint affected (31.7%).

### Time of onset of peripheral arthritis regarding axial disease and regarding axSpA diagnosis

Among the 255 patients who suffered from arthritis at any time, 52 (20.4%) showed the first episode of arthritis before axial symptoms, 35 (13.7%) concomitantly and 168 (65.9%) after axial involvement, respectively. Regarding axSpA diagnosis, 120 (47.1%) showed the first episode before, 15 (5.9%) concomitantly and 120 (47.0%) after axSpA diagnosis, respectively.

### Factors associated with peripheral arthritis

Table 1 shows the baseline characteristics comparing patients with and without peripheral arthritis observed at any time up to the first 5 years of follow-up of the DESIR cohort. Multivariate analysis showed that peripheral arthritis was independently associated with older age (≥ 33 years old, which corresponds to the median age of the DESIR population) [OR 1.60 (95%CI 1.12–2.27)], non-smoking [OR 1.58 (95%CI 1.10–2.27)], HLAB-27 negative [OR 1.47 (95%CI 1.04–2.08)], abnormal CRP values at baseline (≥ 6 mg/dl) [OR 1.99 (95%CI 1.35–2.92)], no history of uveitis [OR 2.03 (95%CI 1.07–3.84)], pervious history of dactylitis [OR 8.50 (95%CI 4.96–14.6)] and previous history of any enthesitis [OR 2.00 (95%CI 1.41–2.84)]. Interestingly, neither gender nor radiographic sacroiliitis were associated with arthritis. Psoriasis showed significant differences in the univariate but not in the multivariate analysis.

**Table 1:** Demographic and clinical features associated with peripheral arthritis in patients with recent onset axial spondyloarthritis

Baseline (M0)	Any arthritis *N* = 255 (%)	No arthritis *N* = 453 (%)	OR (95%CI) Univariate analysis	*p* value*	OR (95%CI) Multivariate analysis	*p* value**
Female gender	147 (57.6)	234 (51.7)	1.27 (0.93–1.74)	0.125		
Age ≥ 33 years old (median age)	148 (58.0)	212 (46.8)	*1.57 (1.15–2.14)*	*0.004*	*1.60 (1.12–2.27)*	*0.009*
Non-Caucasian ethnicity	34 (13.3)	40 (8.8)	1.60 (0.98–2.58)	0.062		
White collar profession	210 (82.4)	387 (85.4)	0.80 (0.53–1.21)	0.280		
High level of education (Univ.)	143 (56.1)	275 (60.7)	0.83 (0.61–1.13)	0.230		
Non-smokers	179 (70.2)	272 (60.0)	*1.57 (1.13–2.17)*	*0.007*	*1.58 (1.10–2.27)*	*0.014*
Alcohol exposure	43 (16.9)	61 (13.5)	1.30 (0.85–1.99)	0.221		
HLAB27 negativity	125 (49.0)	173 (38.2)	*1.56 (1.14–2.12)*	*0.005*	*1.47 (1.04–2.08)*	*0.029*
Absence of MRI-SIJ inflammatory lesions^a^	177 (69.4)	296 (65.3)	1.20 (0.87–1.67)	0.270		
No modified NY criteria^a^	223 (87.5)	373 (82.3)	1.49 (0.96–2.33)	0.075		
CRP ≥ 6 mg/dl	90 (35.3)	105 (23.2)	*1.81 (1.29–2.53)*	*0.001*	*1.99 (1.35–2.92)*	*< 0.001*
Psoriasis	52 (20.4)	66 (14.6)	*1.50 (1.01–2.24)*	*0.047*		
No history of uveitis	238 (93.3)	405 (89.4)	1.66 (0.93–2.95)	0.085	*2.03 (1.07–3.84)*	*0.030*
IBD (Crohn or ulcerative colitis)	10 (3.9)	25 (5.5)	0.70 (0.33–1.50)	0.349		
Dactylitis	76 (29.8)	21 (4.6)	*8.73 (5.23–14.60)*	*< 0.001*	*8.50 (4.96–14.60)*	*< 0.001*
Enthesitis (any location)	174 (68.2)	222 (49.0)	*2.23 (1.62–3.08)*	*< 0.001*	*2.00 (1.41–2.84)*	*< 0.001*
Family history of SpA	102 (40.0)	191 (42.2)	9.91 (0.67–1.25)	0.575		

All results are presented as *n* (%). Percentages indicate number of patients with the covariate from the total number of patients in each category

*mNY* modified New York criteria, *MRI-SIJ* magnetic resonance imaging on sacroiliac joints, *Univ.* university

*p value for the univariate analysis

**p value for the multivariate analysis

^a^mNY criteria and MRI inflammatory lesions according to the local investigator

### Impact on treatment after 5 years of follow-up

Evaluation of the impact of peripheral arthritis on treatment (Table 2) showed that the presence of this clinical feature was significantly associated (*p* < 0.05) with a greater use of drugs such as TNFb [126 (49.9%) vs. 132 (29.1%)], csDMARDs [103 (40.4%) vs. 63 (13.9%)], oral corticosteroids [96 (37.6%) vs. 100 (22.1%)], intramuscular corticosteroids [6 (2.4%) vs. 3 (0.7%)] and intraarticular injection of corticosteroid [79 (31.0%) vs. 31 (6.8%)] during the follow-up. Neither NSAIDs nor intravenous corticosteroids differed between the two groups.

**Table 2:** Impact of concomitant peripheral arthritis on the treatments received during the 5-year follow-up period in patients with recent onset axial spondyloarthritis

	Any arthritis *n* = 255 (%)	No arthritis *n* = 453 (%)	*p* value*
NSAIDs ever	246 (96.5)	435 (96.0)	0.682
TNFb ever	126 (49.4)	132 (29.1)	*< 0.001*
csDMARDs ever	103 (40.4)	63 (13.9)	*< 0.001*
Oral corticosteroids ever	96 (37.6)	100 (22.1)	*< 0.001*
Intramuscular corticosteroids ever	6 (2.4)	3 (0.7)	*0.039*
Intravenous corticosteroids ever	7 (2.7)	7 (1.5)	0.336
Intraarticular corticosteroid ever	79 (31.0)	31 (6.8)	*< 0.001*

All results are presented as *n* (%). Percentages indicate number of patients with the covariate from the total number of patients in each category

*TNFb* TNF blockers, *csDMARDs* conventional synthetic disease-modifying anti-rheumatic drugs

*p value for mixed model with random effects

### Impact on PROs and days of sick leave after 5 years of follow-up

Table 3 represents the impact of peripheral arthritis over 5 years of follow-up through the use of a mixed model with random effects. Patients with arthritis showed higher mean levels of BASDAI over 5 years [40.46 (22.6) vs. 34.30 (20.7); *p* < 0.001]. A total of 128 (50.2%) patients with arthritis and 167 (36.9%) patients without arthritis showed a BASDAI > 40 in a 0 to 100 scale, reflecting an active disease. Patients with arthritis also showed higher mean levels of BASFI [27.89 (23.2) vs. 22.52 (21.0); *p* = 0.001], lower SF-36 score [42.17 (11.7) vs. 43.99 (11.0), *p* = 0.003; and 40.93 (9.65) vs. 43.09 (9.19), *p* < 0.001 for the mental and physical components, respectively] suggesting a worse condition in patients with arthritis, and more days of sick leave [17.97 (57.5) days vs. 12.78 (47.2) days in arthritis vs. no-arthritis patients, respectively, *p* = 0.024] over the 5 years of follow-up than patients without arthritis. Adjusting for TNFb use, BASDAI and BASFI scores remained significantly different between the two groups, as well as BASDAI, BASFI, SF36-MCS and SF36-PCS after adjusting for csDMARDs.

**Table 3:** Impact of concomitant peripheral arthritis on patient-reported outcomes and days of sick leave during the 5-year follow-up period in patients with recent onset axial spondyloarthritis

	Any arthritis *n* = 255 mean (SD)*	No arthritis *n* = 453 mean (SD)	Crude *p* value	*p* value adjusted for TNFb	*p* value adjusted for csDMARDs
BASDAI	40.46 (22.6)	34.30 (20.7)	*< 0.001*	*0.001*	*< 0.001*
BASFI	27.89 (23.2)	22.52 (21.0)	*0.001*	*0.049*	*0.011*
SF36-MCS	42.17 (11.7)	43.99 (11.0)	*0.003*	0.059	*0.022*
SF36-PCS	40.93 (9.65)	43.09 (9.19)	*< 0.001*	0.056	*0.015*
Days of sick leave	17.97 (57.5)	12.78 (47.2)	*0.024*	0.451	0.143

*p* value for mixed model with random effects. All results are presented as mean and standard deviation

*Mean value observed over time

*BASDAI* Bath Ankylosing Spondylitis Disease Activity Index, *BASFI* Bath Ankylosing Spondylitis Functional Index, *csDMARDs* synthetic disease-modifying anti-rheumatic drugs, *SF36-MCS* mental component of the SF36 questionnaire, *SF36-PCS* physical component of the SF36 questionnaire, *TNFb* tumour necrosis factor blockers

## Discussion

The prevalence of peripheral arthritis in axSpA has been reported in different cohorts. However, this is one of the first studies to provide data regarding the natural history of this clinical feature in daily practice. We found that new first episodes of arthritis still appeared over time since the prevalence increased from 21.3% at baseline to 36.0% after 5 years, with a person-time rate of 3.7 new cases per 100 person-years. This last percentage is similar to those reported in non-recent axSpA cohorts [6, 7], suggesting a relationship between the prevalence and the disease duration. Similar results have been recently published concerning peripheral enthesitis in the DESIR cohort, in which the prevalence of this symptom increased from 55.8 to 71% after 5 years of follow-up [19]. This increasing number of new first episode of peripheral arthritis over time is also confirmed by the fact that 65.9% of patients showed the first episode after axial involvement, suggesting that arthritis can appear at any time during the disease. However, we are aware that the low prevalence of peripheral arthritis before axial symptoms (20.4%) could be influenced by “recall bias”, and the higher prevalence of arthritis after axial involvement can be associated with a systematic physical examination by the rheumatologist at every study visit. These data confirm the high probability of occurrence of peripheral arthritis over time after the one of axial symptoms, emphasizing the importance of a systematic iterative check of peripheral manifestations during the monitoring of these patients. Interestingly, 47% of patients showed this first episode after axSpA diagnosis, which means that the information of a past history or current peripheral arthritis might facilitate the diagnosis.

We found that the development of this clinical feature was more common among older patients, confirming the greater prevalence of peripheral arthritis over time. Interestingly, we did not found an independent association with either IBD or psoriasis. The high prevalence of peripheral arthritis in PsA is well known [20]; however, here we observed that in axSpA, this association may not be as evident. Our results showed that the presence of arthritis was also associated with the development of other peripheral symptoms, which are in line with those reported in the ESPeranza cohort, in which dactylitis, arthritis and enthesitis were closely interrelated [21]. Interestingly, we found an inverse association between any concomitant arthritis and (a) HLA-B27 positivity, (b) presence of uveitis and (c) tobacco use. All these associated factors are in the opposite direction than the ones reported in patients with a pure axial disease (e.g. B27 positivity, uveitis and smoking habits) [22], suggesting a different physio pathological pathway. In addition, the relatively low prevalence of MRI or X-ray sacroiliitis could indicate that part of these patients has predominant peripheral disease instead of axial disease. However, all patients in the DESIR cohort have suffered from IBP suggestive of axSpA at any time, which leads to their classification as axSpA. An inverse association between smoking and peripheral arthritis in SpA patients has been previously reported [23, 24]. A recent study also suggested the “smoking paradox” in PsA, which described that smoking increased risk of PsA in the general population but appeared protective among psoriatic patients [25]. In this study, authors raised a possible bias by uncontrolled confounding. Regarding our results, we wondered whether non-smoking and peripheral arthritis are biologically or statistically associated, so further studies are needed to evaluate this association.

Concerning drug intake, we found that patients with arthritis had a greater use of csDMARDs and glucocorticoids. These results are not surprising since in accordance to the current ASAS-EULAR recommendations, glucocorticoids injections and sulfasalazine may be considered in case of peripheral arthritis [26]. However, it is interesting that, in our study, patients with arthritis showed a greater use of bDMARDs but similar use of NSAIDs compared to that of patients without arthritis. One may speculate that NSAIDs would control the axial symptoms in both groups, but the presence of arthritis could increase the likelihood of starting a TNFb because of the higher disease activity and the negative impact of peripheral arthritis on PROs. In fact, a previous study in the DESIR cohort has demonstrated that the Ankylosing Spondylitis Disease Activity Score (ASDAS) and the presence of synovitis act as independent factors associated with TNFb initiation [27].

Indeed, here, we demonstrated that patients who have ever suffered from peripheral arthritis at any time during the 5 years of follow-up period showed poorer quality of life and increased days of sick leave over time. Despite the evaluation of the clinical relevance of these results is more difficult, the fact that 36.9% of patients without arthritis and 50.2% with arthritis showed an active disease (BASDAI > 40) demonstrates the importance of this feature.

One limitation of this study is the difficulty of precisely evaluating arthritis that occurred before the inclusion visit and between two study visits because of the absence of physical examination in these time points. However, we have taken into account patients who received corticosteroid intraarticular injections between two visits to be considered as patients with arthritis. Another limitation is that we could not definitively rule out the possibility of a concomitant osteoarthritis in certain patients, partly because intra-articular injections could be performed for this diagnosis in some cases. The main strength is that this report is one of the few non-RCT studies that evaluate this feature over time.

## Conclusions

In summary, in this study, we observed that peripheral arthritis can appear at any time during the disease and has a high burden of disease (deteriorating quality of life and causing days of sick leave). This finding is the reason why rheumatologists should check systematically this clinical feature during the monitoring of these patients. Other studies are required in order to confirm or not these results and to better understand the underlying pathological process.

## Data Availability

Data are available from the authors upon request with permission of the DESIR cohort scientific committee.

## Abbreviations

AS: Ankylosing spondylitis
ASAS: Assessment in SpondyloArthritis International Society
axSpA: Axial spondyloarthritis
BASDAI: Bath Ankylosing Spondylitis Disease Activity Index
BASFI: Bath Ankylosing Spondylitis Functional Index
bDMARDs: Biological disease-modifying anti-rheumatic drugs
CRP: C-reactive protein
csDMARDs: Synthetic disease-modifying anti-rheumatic drugs
IBD: Inflammatory bowel disease
IBP: Inflammatory back pain
MRI: Magnetic resonance imaging
NSAIDs: Nonsteroidal anti-inflammatory drugs
OR: Odds ratio
PROs: Patient-reported outcomes
PsA: Psoriatic arthritis
pSpA: Peripheral spondyloarthritis
SF36-MCS: Mental component of the SF36 questionnaire; SF36-PCS: Physical component of the SF36 questionnaire
SpA: Spondyloarthritis
